# Immuno-stimultory/regulatory gene expression patterns in advanced ovarian cancer

**DOI:** 10.18632/genesandcancer.78

**Published:** 2015-09

**Authors:** Kevin H. Eng, Isabelle Weir, Takemasa Tsuji, Kunle Odunsi

**Affiliations:** ^1^ Department of Biostatistics and Bioinformatics, Roswell Park Cancer Institute, Buffalo, NY, USA; ^2^ Center for Immunotherapy, Roswell Park Cancer Institute, Buffalo, NY, USA

**Keywords:** cancer testis antigen, co-stimulation, gene expression, ovarian cancer

## Abstract

It has been established that a high degree of tumor-infiltrating T cells is associated with ovarian cancer prognosis. We hypothesized that tumors display an immune-related program of transcription that can act in a stimulatory or a regulatory manner. We analyzed transcriptome-wide gene expression data from 503 ovarian tumors from the Cancer Genome Atlas to identify genes that show differential prognoses when stratified by CD3 expression. Genes with immunological functions and tumor antigen genes were selected for analysis. We repeated our analysis in an independent validation study. Five genes showed stimulatory/regulatory patterns at a high level of confidence (Bonferroni p < 0.05). Three of these (MAGEA8, MPL, AMHR2) were validated and one (WT1) could not be evaluated. These patterns show specific prognostic effect only in conjunction with CD3 expression. When patients express multiple transcripts in poor prognosis directions, there is a dose response: increasingly regulatory type tumors are associated with higher stage, lower treatment response and shorter overall survival and progression free survival. The high-confidence set of transcripts (MAGEA8, MPL, AMHR2, WT1) and selected low-confidence hits (EPOR, TLR7) alone or in combination represent candidate prognosis markers for further investigation.

## INTRODUCTION

There is significant evidence that intra-tumoral CD8+ cytotoxic T lymphocytes (CTL) are beneficial for prognosis in ovarian cancer [1] as well as other cancer types. However, it is known that the active intervention of immunosuppressive T cells (Tregs) reverses the prognostic importance of CTLs [2]. The combination of signals leading to the suppression of immune responses at the tumor site is only partially resolved. We know that immunosuppression requires T cell receptor (TCR)-mediated antigen-specific activation and this individual-specific expression of a wide array of immunogenic tumor antigen has hampered characterization of antigen-specific T-cell responses in the tumor site.

On the patient level, high-throughput genomic investigation may yield clues for further immunological studies. For example, Leffers and colleagues [3] stratified ovarian tumors based on histopathologically con-firmed low and high CTL infiltration and employed expression arrays to look broadly at almost 16,000 unique transcripts measured from whole tumor tissue. The study identified 54 genes associated with disease-specific survival and CTL infiltration, confirming their relevance by further immunohistochemistry. In an immunological and systems biology theme, genomic studies characterized the T cell receptor (TCR) induced intracellular signaling response evident in gene expression from purified T cell populations [4], [5] and CD4+CD45RA+ T cells [6]. These studies suggest that genomic-scale tools can be brought to bear to answer immunological questions using clinical data supported by standard immunological techniques.

Currently, bioinformatic workflows either focus on one gene at a time or associate sets of genes prioritizing correlation over biology. In contrast, immunologic processes often rely on complex interactions of specific genes. For example, in addition to regulatory effects that promote the termination of response, the two signal model of T-cell co-stimulation suggests that a complicated interaction of several genes is involved in the activation of the adaptive immune response [7], [8]. Sharpe [9] reviews the various members of the CD28 receptor family and their matching ligands noting both ICOS and CTLA-4 to be co-stimulatory and regulatory respectively. Thus, finding a way to uncover these effects using expression data with the right statistic is an important goal.

New genomic study associations that carefully model multiple genes will have strong relevance for immunotherapy [10]. In particular, we focus on cancer testis (CT) antigens [11] and a list of expert-prioritized antigens [12] all of which have clear prognostic value will aid the identification of targets for immunotherapy. Both selective targeting of CD28 co-stimulatory family members [13] and the blockade of immune checkpoints (i.e., regulatory genes) [14] are therapeutic strategies currently under investigation in many cancers.

To augment genomic studies, we hypothesized that co-stimulatory and regulatory effects may be seen at the patient level by ordering patient prognoses according to the expression of a marker of activation and a candidate regulatory transcript. That is, we imagine that we might stratify patients by a transcript-based score, like the 54 CTL markers, in order to look for transcripts that are associated with further modification of the prognostic CTL effect. While an immunological study is typically focused on the careful characterization of cell populations, a genomic-view tends to be limited to whole tumor tissues subject to cellular and genetic heterogeneity [3]. We conjecture that the tradeoff will favor discovery of new prognostic associations for further study with formal immunological methods.

In this article, we define a set of co-stimulatory/regulatory hypotheses and the test statistics that will summarize the evidence for these models using gene expression and progression-free survival. Using the CD28-CD80 (B7-1) family as a positive control, we show that this method can identify canonical co-stimulatory signaling in the Cancer Genome Atlas (TCGA) study of high-grade serous ovarian cancers [15]. We then examine the family of CT antigens for co-stimulatory and regulatory effects and study the co-expression patterns of selected genes.

## RESULTS

### CD28 family stimulatory/inhibitory system recapitulated

The CD28/B7-1 signaling family is a model co-stimulatory system for T cell activation [16]and includes the ICOS receptor and its ligand ICOSL [13]. When added to a TCR-mediated activation signal, ligation of the ligand CD86 to its CD28 family receptor, CTLA-4, inhibits T cell response [14]. Per our hypothesis, these transcripts should be associated with a pattern of PFS in T-cell infiltrated tumors.

**Figure 1:**
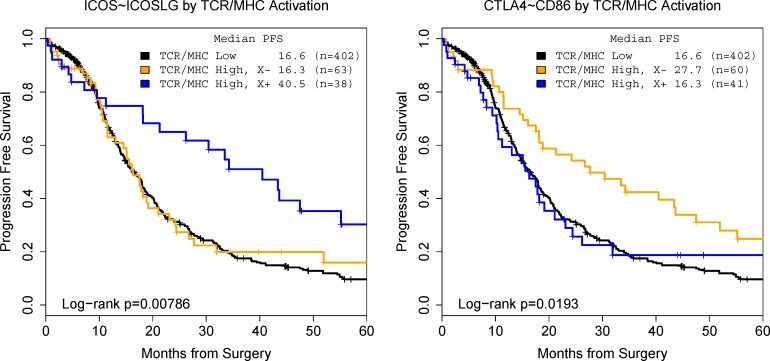
Model co-stimulatory/regulatory effects can confirmed by studying patient prognosis ICOS∼ICOSLG is a co-stimulatory effect (left) and CTLA4∼CD86 is a regulatory effect (right).

To quantify activation, we characterized major histocompatibility complex class I (MHC-I) and TCR activation using HLA-A and CD3 transcript expression. The co-expression of CD3 and MHC class I itself is associated with better PFS (Cox model: HR=0.636, p=0.012). We consider patients with the highest activation level (top 20%) and find a clinical difference in median PFS of 16.6 versus 19.1 months (log-rank p=0.0263). In contrast, in a CD3/MHC-I co-expression group, we confirmed that co-expression between CD28 family member ICOS and its ligand (ICOSLG) is associated with a beneficial prognostic effect (Figure 1): co-expression of ICOS and ICOSLG is a stimulatory marker (Table 1). When split at the median signaling marker level, patients with high activation and stimulatory signals have better PFS than patients with low activation or low co-stimulatory signals (16.4 versus 40.5 months, p=0.0019). This is consistent with the expected function of the ICOS receptor [9].

**Table 1:** Patterned hypotheses for marker (M) and regulator (R) reflecting improved (+) and baseline (−) survival

Type of co-regulation	M−	M+R−	M+R+	Interpretation
Stimulatory	−	−	+	Regulator is required for Marker+ effect
Regulatory	−	+	−	Regulator reverses the effect of Marker+

Similarly, we confirmed that transcript association between CTLA-4 and CD86 is associated with a regulatory effect. Patients with high CD3/MHC-I co-expression but low CTLA-4 mediated signals have a better prognosis than patients with high receptor activation and high CTLA-4 expression (27.7 months versus 16.6, p=0.0193). Together, these two results confirm that our statistical technique can identify stimulatory and regulatory effects on T cell mediated anti-tumor activities that affect patient prognosis based on stratification by CD3 transcript levels. Subsequently, we will use this technique in an exploratory manner.

### Search of immune genes and antigens reveals candidate co-stimulatory and regulatory actors

We performed a global search for stimulatory and regulatory effects in 510 selected genes spanning immunologically relevant sets, which are described in the methods section. Considering above median expression of CD3 as the stratifying marker, the strongest associations that we observed (Bonferroni p< 0.05) suggest FAS and MAGEA8 have regulatory effects and MPL, AMHR2 and WT1 have stimulatory effects; a total of 47 genes have regulatory and 62 stimulatory effects at FDR< 0.05.

In Table 2, we list the effects of the 5 Bonferroni significant genes as well as the two largest effects from all 109 FDR significant genes (EPOR and TLR7); the former are strong confidence results and the latter are weaker, both are adjusted for multiple comparisons. For these genes, the effect of marker expression on PFS is minimal in a group with low CD3 expression. In contrast, expression of the same marker has a high CD3-specific effect on PFS. These effects are either protective or deleterious per the stimulatory/regulatory designation (Table 1) supporting the hypothesis that these molecules function through regulating T cell mediated anti-tumor activities.

**Table 2:** Significant co-stimulatory/regulatory effects (Bonferroni p < 0.05 or large effect and FDR< 0.05) and independent data validation status Bold text highlights the specific hypothesis. WT1 was not measured in the validation data.

			Median Months PFS				Discovery Adjusted		Validation Adjusted
			CD3 Low		CD3 High				
Gene		% High	Low	High	Low	High	p-value	FDR	p-value
Regulatory	MAGEA8	50%	13.8	17.4	26.8	15.4	0.0266	0.0133	0.0291
	FAS	40%	14.1	17.9	23.8	16.1	0.0112	0.0111	0.3081
	TLR7	70%	14.8	14.9	34.0	18.0	1.0000	0.0499	0.0286
Stimulatory	MPL	70%	15.1	14.8	15.4	24.2	0.0456	0.0153	0.0155
AMHR2	WT1	70%	14.6	14.8	13.0	22.3	0.0134	0.0067	NA
	AMHR2	50%	13.8	17.4	14.7	25.1	0.0071	0.0067	0.0038
	EPOR	20%	14.0	16.4	18.0	40.1	0.5330	0.0323	0.0206

The threshold for high expression was determined for each marker separately as described in the Methods section and is given in the percentage column: the top 20% of EPOR expressing patients were considered high while 70% of patients are designated as high TLR7 expressers. Multiple comparisons to identify these levels are accounted for in the p-value calculations.

**Figure 2:**
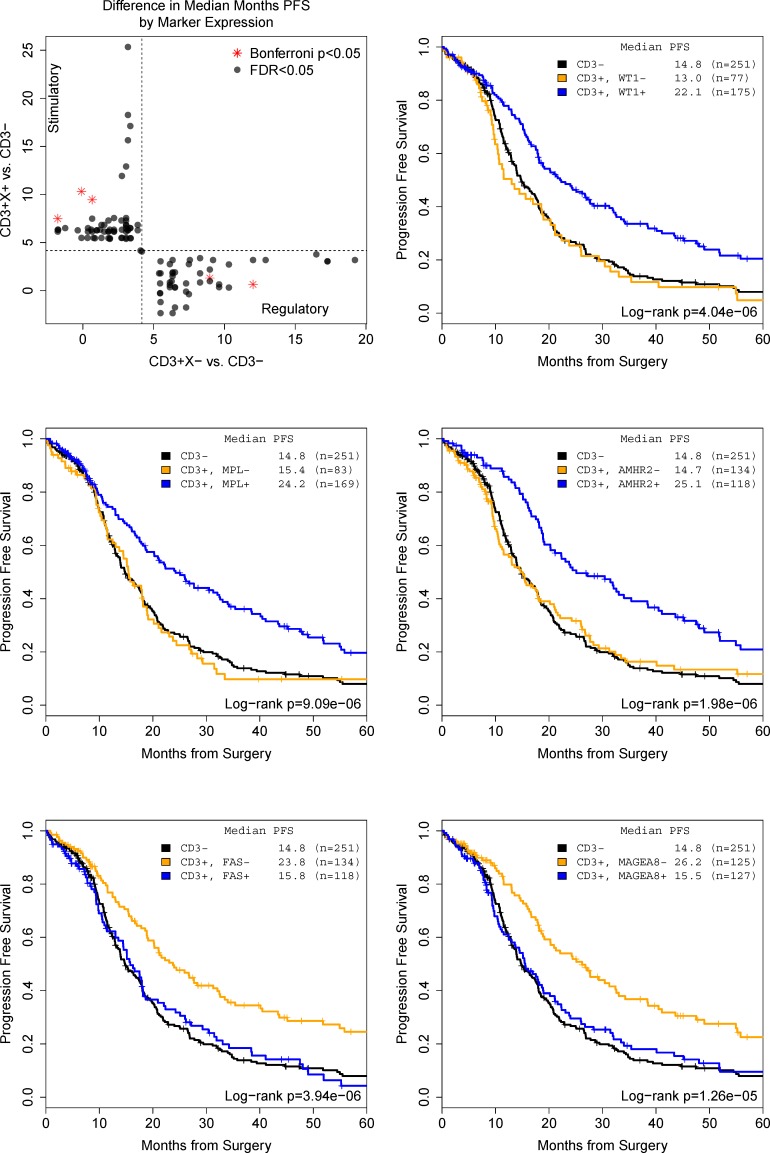
Change in progression-free survival between CD3− and CD3+ subgroups stratified by candidate markers (Top left) uncovers five high confidence (Bonferroni p < 0.05) genes with both regulatory and stimulatory effects

We present survival curve plots (Kaplan-Meier estimates) for the five strong confidence genes in Figure 2. In these plots, the dark curves reflect prognosis for all CD3 low patients and the colored curves for CD3 high patients. The orange curve shows low candidate gene expression and the blue high expression.

As shown in Figure 2, high expression of stimulatory markers (WT1, MPL, and AMHR2: blue lines) or low expression of regulatory markers (FAS and MAGEA8: orange lines) significantly prolonged PFS in a group with T cell-infiltrated (CD3 high) tumors while PFS of the complementary group (low stimulatory, high regulatory marker expression) overlaps the CD3 low group. In all cases, the single gene indicates a sub-population of at least 15% of the study whose prognosis differs by about 10 months following a stimulatory or regulatory pattern.

### Validation via independent study with immunohistochemistry data

An independent dataset of microarray-based gene expression in advanced ovarian tumors (n = 110) was used as a validation set 3. For this study, the number of CD3+ infiltrates as determined by immunohistochemical staining was used to stratify patients as a more accurate marker for the number of TILs than array-based CD3 transcript level. In the validation dataset, MAGEA8, TLR7, MPL, AMHR2, EPOR show Bonferroni significant expression values in a direction consistent with the discovery data set. WT1 was not measured on the array used in this study and we failed to validate FAS expression.

To rule out whether these significant findings can be traced to prognostic value independent of CD3 expression, we regressed each gene individually on PFS in both data sets. We found no significant association (Bonferroni p< 0.05 or FDR< 0.05) between PFS and any single gene. This suggests that the stimulatory and regulatory effects are uncovered only when considering tumors with high infiltration of CD3+ T cells.

### Joint modeling and co-expression of markers

We tested whether the multiple gene markers redundantly identify the same set of patients and found that no single combination of high expressing genes was unusually prevalent. So, we analyzed patients aggregated by the number of regulatory and stimulatory genes that were highly expressed.

In a high CD3 group, on average, each patient expresses high levels of 1.98 stimulatory markers and high levels of 1.79 regulatory markers on average. Table 3 captures the variation in expression of each set of markers. Each regulatory marker increases PFS hazard (HR=1.46) when it is highly expressed and each stimulatory marker decreases hazard (HR=0.78) when expressed; this effect is consistent with both the discovery and validation sets. These hazard ratios translate to significant variation in clinical prognosis: 55 to 11 months PFS across the range of observed regulatory markers and 11 to 77 months PFS for stimulatory markers.

**Table 3:** Distribution and effects of high expression of markers among high CD3 patients only

	Regulatory	Stimulatory
Mean # High	1.79	1.99
Median # High	2.00	2.00
HR per +1 High (95% CI)		
Discovery	1.46 (1.28-1.66)	0.78 (0.72-0.85)
Validation	1.49 (1.16-1.84)	0.82 (0.67-0.99)
Median PFS		
0 High	54.9 (n= 18)	11.2 (n=20)
1 High	27.7 (n= 63)	10.7 (n=63)
2 High	18.2 (n=126)	19.1 (n=79)
3 High	10.5 (n= 45)	35.4 (n=80)
4 High		76.9 (n=10)

### Clinical associations with regulatory and stimulatory expression

When considered jointly in a multivariate model, no one marker stood out with a dominating prognostic effect suggesting a highly heterogenous process (data not shown). As patients most frequently have high expression of both regulatory and stimulatory genes, we hypothesized the prognostic effect of an imbalance in the number of regulatory effects and stimulatory effects. We classified patients by whether they expressed more stimulatory or regulatory antigens (ties were classified as more regulatory) and considered the association of these sets with clinical and pathological variables (Table 4).

**Table 4:** Association between stimulatory or regulatory expression and clinical and pathological variables Totals may not sum to n due to missing data.

Discovery Set	More Stimulatory	More Regulatory	Low CD3/TIL	p-value
n	109	143	251	
Age Mean	58.3	61.4	59.5	0.0755
Stage				
I/II	9	11	18	0.9277
III/IV	99	132	233	
Grade				
G1/G2	7	14	45	0.0046
G3/G4	99	127	201	
Treatment Response				
Complete	77	73	138	0.0261
Partial/Stable/Progressive	18	40	63	
Debulking Status				
Suboptimal	68	99	181	0.4226
Optimal	26	26	50	
Discovery set				
OS median months	65.0	35.3	38.4	<0.0001
PFS median months	38.4	14.5	14.8	<0.0001
Validation set				
OS median months	12.0	25.0	11.0	0.0002
PFS median months	9.0	14.0	6.0	<0.0001

We note that more stimulatory cases tended to be higher grade (93% vs. 85%, p=0.0046) and to respond better to primary treatment (81% vs. 67%, p=0.0261). As expected, these cases were also associated with longer PFS and OS. In the validation set, we note that imbalances predicted the opposite OS and PFS pattern: patients with regulatory effects had better survival outcomes. Given that the counts themselves had a consistent direction in Table 3, this may be due to the small sample size or selection bias based on the added requirement for tumor-infiltrating CD3+ T cell count and pathological review.

## DISCUSSION

We have conducted a computational study of gene expression patterns associated with prognosis of advanced ovarian cancer patients by re-analyzing data from the Cancer Genome Atlas. Using our method stratifying patients by tumor-infiltrating CD3+ T cells, we identified 5 high confidence genes and 2 genes with weaker confidence but large, significant clinical effects that positively or negatively associated with prognosis. Importantly, expression of these genes showed little prognostic impact on patients whose tumor was not highly infiltrated by CD3+ T cells. Therefore, these candidate genes are considered to regulate anti-tumor T-cell responses at the local ovarian tumor site and are promising targets in developing efficient immunotherapeutic strategy in a personalized fashion.

Inferentially, these effects are discovered only with the a priori knowledge that they are immune-related genes that may be related to cancer prognosis; we believe this approach is a powerful way to identify candidate markers for further study and we review the top hits here noting that they all have existing independent literature strengthening our findings.

WT1 was rated the most important antigen by experts in immunotherapy on the basis of its fair therapeutic function, demonstrated immunogencity, oncogenicity and high specificity, expression in cancer and stem cells, prevalence of antigen-positive cancers, multiple epitopes and internal localization of expression. It is expressed in ovarian cancer, but has conflicting reports about its value as a prognostic marker [17], [18]. We found the prognostic value of WT1 requires the presence of tumor-infiltrating CD3+ T cells: the WT1 antigen appeared with a strong stimulatory effect (for nearly 70% of patients, 9-10 months of improved PFS when co-expressing high levels of CD3). Unfortunately, we could not validate the effect in the independent data because the gene was not interrogated.

MAGEA8 is a CT antigen whose expression in normal adult tissues is restricted to the testis and placenta [19]; family members MAGEA3, MAGEA1 were also scored by immunotherapy experts (ranks 8 and 44 out of 75) and have been shown to be expressed in ovarian cancers [20]. In the TCGA study, below median MAGEA8 expression was associated with 11.4 months increased PFS making, it the strongest verifiable effect. It is interesting to note that in contrast to WT1, high expression of MAGE A8 was associated with poorer PFS in patients with high CD3 tumors, potentially indicating an immunosuppressive role of MAGEA8 such as via activation of immunosuppressive Tregs.

A high-confidence finding, the anti-mullerian hormone receptor (AMHR2, MISIIR), which is a member of the TGFB receptor family of serine/threonine receptor kinases, is expressed in 6 human ovarian cancer cell lines [21], [22] in vitro treatment with MIS led to decreased colony formation consistent with the co-stimulatory effect we observed here: above median expression of AMHR2 leads to 10.4 months increased survival.

TLR7 is a low confidence hit (FDR< 0.05), but it had the largest regulatory effect: the lowest expressing 30% of patents had an increase of 16 months PFS in the CD3 high group. Ligands that activate the toll-like receptors are attractive as candidate adjuvants that activate the innate immune cells including antigen-presenting cells for immunotherapy [23]. Although potential therapeutic benefit was reported for a TLR7 agonist [24] used in heavily pre-treated patients, the roles of TLR7 expression in ovarian cancer cells requires further investigation [25]. Identification of TLR7 as a regulatory marker in the CD3 high group indicates an immunosuppressive phenotype of TLR7-expressing tumor.

Thrombopoietin receptor MPL (TPO-R) is a proto-oncogene whose ligand (TPO) has been recently promoted as a marker for ovarian cancer and with existing agonists [26]: found higher levels associated with more advanced cases and levels of serum thrombopoietin decreased after primary treatment. In ovarian cancer patients, the level of TPO is linked to thrombocytosis and to advanced disease and poor survival [27]. The receptor itself may be a marker for patients likely to avoid chemotherapy-induced thrombocytopenia [28] however in vitro studies have found conflicting levels in ovarian cell lines [29], [30]. In our study, we noted MPL expression in the top 70% of patients was associated with an 8.8 month increase in PFS. Interestingly, one low-confidence hit is another hematopoietic receptor: the erythropoietin receptor (EPOR, FDR= 0.0323, validation Bonferroni p = 0.0297). Again EPOR has an immuno-stimulatory effect: the top expressing 20% of patients gain nearly 22 months of PFS. Adverse effects of erythropoiesis-stimulating agents have been reported [31], underscoring the point that this computational approach may be sensitive and powerful but is incomplete in a clinical setting.

A weakness of the array-based approach is that it is unclear to what degree the expression levels translate to clinically familiar ways to detect expression (RT-PCR and IHC). A natural next step is to assay primary tumors to determine the prevalence of protein expression of these markers. Thresholds are based on the observed samples so further work is required to calibrate the reference ranges to normal tissues or cancer samples.

## METHODS

### Expression databases and preprocessing

We downloaded data for the TCGA study [15] Affymetrix HT HG-U133A arrays only. The values were quantile normalized and background subtracted in the standard RMA workflow and values were converted to an empirical quantile scale for interpretability. Probe sets mapping to the same gene were summarized by a brightest spot rule and genes were matched across studies by standard HUGO gene name.

The data from the Leffers and colleagues study [3] array annotation, protocol and data are available from the Gene Expression Omnibus (GEO). These are a subset from GEO:GSE13876 [32] assayed on an Operon two color oligonucleotide array (GEO:GPL7759). The original study noted that disease-specific survival is higher for CD8+ tumor infiltrating lymphocytes (TILs) so this forms a positive control dataset with gold standard classification (i.e., immunohistochemistry) for CD8+ TILs. Note that the original study had 54 samples with TIL information; we obtained TIL counts for 110 cases from the study authors (personal correspondence, Ate G. J van der Zee).

### Patients and clinical datasets

We define progression-free survival (PFS) as the time from surgery to death or progression (for patients with no disease-free interval after primary therapy) or time from surgery to death or recurrence (for patients with a disease-free interval). Throughout this article, we consider PFS to be the endpoint of interest because it reflects a clinical decision point for advanced ovarian cancer (whether to reapply platinum-based chemotherapy). Overall survival (OS) is omitted because post-recurrence treatments for ovarian cancer are varied and not reported.

As described in their original article [15], the TCGA ovarian patients are all high-grade serous ovarian cancer treated surgically with adjuvant platinum chemotherapy in conjunction with a taxane chemotherapy (94%). We removed a small number of patients treated with neoadjuvant therapy. The median age is 59.8 years and 19.3% are suboptimally debulked (>2cm) with 22.7% achieving full resection (no macroscopic disease). TCGA protocol stated that all patients received adjuvant platinum/taxane chemotherapy. The whole study had a median OS of 44.1 months (39.6-47.7, 95%CI), PFS of 17.5 months (15.4-18.5), and a 5-year survival rate of 31.3% (26.5%-37.0%).

A subset of a larger ovarian cancer study of patients treated at University Medical Center Groningen (UMCG, Netherlands) between 1990-2003 [32], validation dataset [3] is defined by patients with clinical, survival and immunohistochemistry data. Per the original articles, these patients are all advanced (Stage III, IV) serous ovarian cancer. The median age is 60 years and 64% of the patients were suboptimally debulked (> 2cm). All patients received adjuvant platinum-based chemotherapy and 35.1% also received taxane. The subset has a median OS of 12 months (12-16), PFS of 8 months (7-10) and 5-year survival rate of 12.2% (7.1%-21.0%). Differences in survival might be attributed to different practices between the countries and over time.

### Selected immune gene list

We selected genes comprising the immunotherapeutic target antigens selected by immunotherapy experts [12] (number of genes, n=49), antigen processing and presentation genes (KEGG:hsa04612, n=58), cytokines and their receptors (KEGG:hsa04060, p=229), cluster of differentiation genes (n=61), cancer testis antigens (CTdatabase [11], n=92), T-cell receptor signaling genes restricted to the cell surface (KEGG:hsa04660, n=15) and Toll-like receptor genes (KEGG:hsa04620, n=15). The overlap between genes is minimal: just 11 genes are common to two pathways. The supplementary materials contain the complete list of genes and their subcategorization (Table S1), selected CT antigen information (Table S2), the antigen list (Table S3) and the list of validated results (Table S4).

### Gene expression markers and stimulatory/regulatory transcript patterns

We define stimulatory/regulatory transcripts to have a patterned effect on progression free survival. In particular, Table 1 uses + and − to represent improved and baseline median PFS times and M and R to mean the marker and regulator. (In the validation data set, M is the TIL count.) The hypothesis that the + and − groups differ can be tested directly using a two sample log-rank tests with the appropriate statistical contrast.

Because these are continuous expression markers (array-based), for the TCGA data, we choose a dichotomizing point based on searching through quantiles (20% 30%, …, 80%) and recording the best split by log-rank p-value. The final p-value is adjusted for multiple comparisons including the number of tested quantiles (Bonferroni for family-wise error rate control; Benjamini and Hochberg [33] for FDR control).

## SUPPLEMENTARY TABLES


